# Reaktivierung eines okulären Schleimhautpemphigoids im Rahmen von Zoster ophthalmicus und COVID-19

**DOI:** 10.1007/s00347-023-01861-1

**Published:** 2023-05-09

**Authors:** Sarah Stanischewski, Arne Viestenz, Jens Heichel

**Affiliations:** Universitätsklinik und Poliklinik für Augenheilkunde, Universitätsklinikum Halle (Saale), Martin-Luther-Universität Halle-Wittenberg, Ernst-Grube-Str. 40, 06120 Halle (Saale), Deutschland

## Anamnese

Eine 80-jährige Patientin stellte sich erstmalig mit Epiphora, Trichiasis und Bindehauthyperämie vor. Der Visus lag bei Handbewegungen rechts und 0,16 am linken Auge. Eine laterale Zügelplastik am linken Auge sei bereits vor mehreren Jahren ex domo erfolgt.

Anamnestisch waren an Vorerkrankungen ein Vorhofflimmern, eine Hypercholesterinämie, Mammakarzinom (chemotherapeutisch und operativ versorgt) und eine Niereninsuffizienz bekannt. Rheumatologische oder dermatologische Erkrankungen verneinte die Patientin. Es waren keine Allergien bekannt, und zum Zeitpunkt der Erstvorstellung wurden keine Augentropfen angewandt.

**Figure 1:**
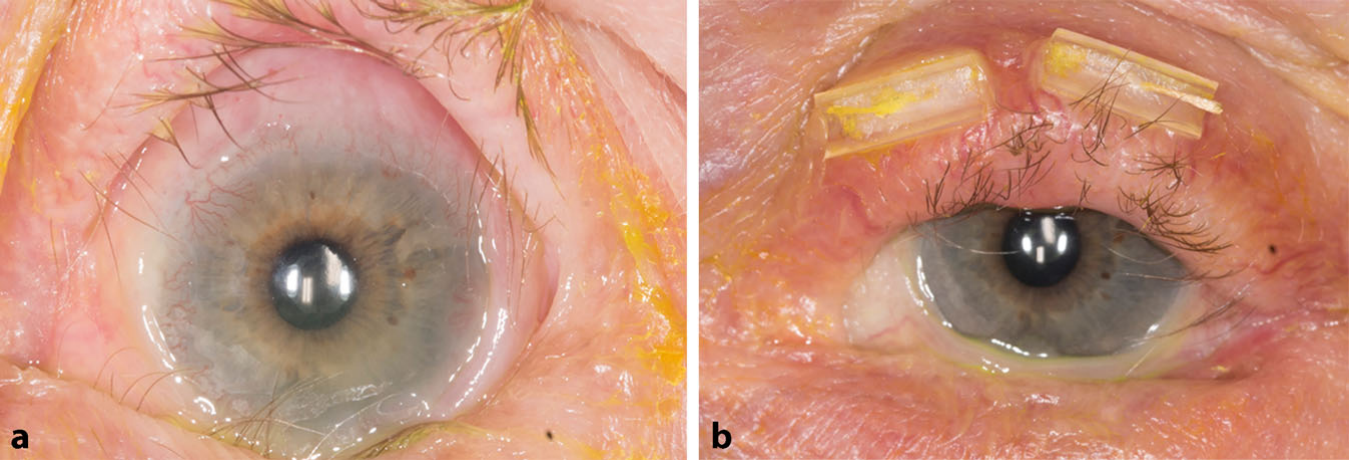

Bei auffälliger Fornixverkürzung und Symblephara (Abb. 1) erfolgte eine umfassende Abklärung der Verdachtsdiagnose eines okulären Schleimhautpemphigoids (OSP). Nach Mondino und Brown lag klinisch am rechten Auge bereits bei Erstvorstellung das Stadium IV und am linken Auge lag das Stadium II–III vor. Serologische Untersuchungen ergaben keinen Autoantikörpernachweis. Es lagen keine extraokulären Manifestationen eines Pemphigoids vor. Im Verlauf erfolgte zudem eine Diagnosesicherung mittels Bindehautbiopsie.

Die Patientin erhielt initial 50 mg Prednisolon, und es wurde eine Lokaltherapie mit Ciclosporin 2 % begonnen. Im Verlauf wurde das Prednisolon auf eine Erhaltungsdosis von 5 mg reduziert und zusätzlich eine Systemtherapie mit Mycophenolat-Mofetil 1500 mg in Rücksprache mit den Rheumatologen und Dermatologen täglich begonnen. Nach medikamentöser Einstellung und einer Lidrekonstruktion nach Ohashi [1] am linken Auge bei entropiumbedingter Trichiasis kam es zum Visusanstieg auf 0,4 links und zu einem reizfreieren Befund (Abb. 1). Postoperativ zeigte sich eine Befundstabilisierung. Ein Jahr nach Einleitung der Systemtherapie des OSP erkrankte die Patientin jedoch innerhalb von 2 Monaten an COVID-19 und danach an einem Zoster ophthalmicus.

## Befund

**Figure 2:**
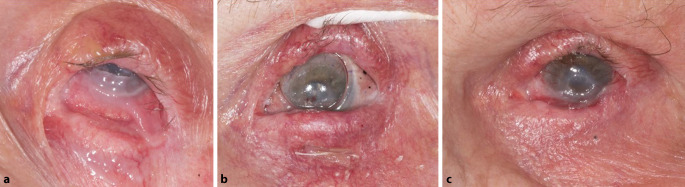

Es folgte eine Reaktivierung der Erkrankung mit v. a. am linken Auge beginnendem Ankyloblepharon sowie vaskulärem Pannus der Hornhaut mit Ulkus (Stadium IV nach Mondino und Brown) (Abb. 2). Es zeigte sich zudem eine erneute Lidfehlstellung mit Entropium des Ober- und Unterlides. Der Visus nahm links auf die Wahrnehmung von Handbewegungen ab, rechts auf lux projectio recta. Das Mycophenolat-Mofetil wurde während der akuten Infektionsphase auf 1000 mg täglich reduziert. Die Tensiowerte waren palpatorisch normoton. Fundoskopisch zeigte sich eine senilatrophe Papille bei Netzhautanlage am linken Auge, rechts lag sonographisch eine Netzhautanlage vor.

## Diagnose

Es wurde die Diagnose eines reaktivierten okulären Schleimhautpemphigoids (Stadium IV nach Mondino und Brown) nach einer Zoster-ophthalmicus- und COVID-19-Erkrankung gestellt.

## Therapie und Verlauf

Nach Abwarten des akut inflammatorischen Prozesses wurden die topische und systemische Therapie optimiert. Es erfolgten eine Symblepharotomie, Pannektomie, Mitomycin-C-Anwendung und eine Fornixexpansion mit Amnionmembrantransplantat und nahtfixierter Illig-Schale. Neben der Dauertherapie mit Ciclosporin 2 % und Mycophenolat-Mofetil wurde eine intensive Lokaltherapie mit Tränenersatzmitteln und glukokortikoidhaltigen Augentropfen veranlasst. Es erfolgte zudem eine antibiotische Abschirmung mit konservierungsmittelfreien Ofloxacin-Augentropfen. Postoperativ kam es zu einem Visusanstieg auf Fingerzählen und Befundverbesserung. Die Patientin berichtete vom Wiedergewinn ihrer Orientierungsfähigkeit.

Im weiteren Verlauf entwickelte die Patientin jedoch eine Limbusstammzellinsuffizienz mit weiterem Progress des Schleimhautpemphigoids samt erneuter Symblepharonbildung. Auch eine erneute Fornixexpansion konnte keine Visusbesserung mehr erzielen. Das Follow-up liegt bei 1,5 Jahren.

## Diskussion

Das okuläre Schleimhautpemphigoid (OSP) stellt eine chronisch fortschreitende Autoimmunerkrankung mit progressiver Bindehautvernarbung dar, die auf einer Autoimmuntyp-II-Überempfindlichkeitsreaktion beruht [2]. Viruserkrankungen können eine Erstmanifestation oder wie in diesem Fall eine Reaktivierung bestehender Pemphigoiderkrankungen bedingen [3].

Aufgrund der immunsuppressiven Systemtherapie stellen Patienten mit OSP eine Hochrisikogruppe für Infektionskrankheiten dar [4]. Die genaue pathogene Beziehung zwischen OSP und viralen Infektionen ist jedoch nicht vollständig verstanden. Es werden verschiedene Mechanismen, die sowohl genetische Faktoren als auch Umwelteinflüsse umschließen, diskutiert. Die 4 meistdiskutierten Hauptmechanismen sind dabei die molekulare Mimikry (Nachahmung molekularer Strukturen des Wirts), eine „bystander activation“ (akute Infektion bewirkt Aktivierung benachbarter T‑Zellen), die Aktivierung von T‑Zellen durch Superantigene und das „epitope spreading“ (Immunantwort weitet sich über das erkannte Epitop eines fremden Antigens aus) [5].

In der Pathogenese des OSP ist die Bildung von Autoantikörpern gegen Proteine innerhalb der Basalmembranzone entscheidend. Zu diesen Autoantikörpern gehören u. a. BP180, α6β4-Integrin, BP230 oder Laminin 332. Der Serologie kommt in der Diagnosestellung in der Hinsicht eine besondere Bedeutung zu, da insbesondere Patienten mit Antikörpern gegen Laminin 332 ein erhöhtes relatives Risiko für solide Krebserkrankung aufweisen [6]. Auch auf die Autoantikörperproduktion scheinen virale Erkrankungen Einfluss zu nehmen. Kamya et al. konnten bereits zeigen, dass eine VZV-Infektion die Antikörperproduktion von BP180 steigern kann [7].

Auch beim SARS-CoV-2-Virus ist bereits bekannt, dass es Autoimmun- und rheumatische Krankheiten über die oben genannten Mechanismen bedingt und ein hohes Maß an molekularer Mimikry zwischen SARS-CoV-2 und BP180 vorliegt [5, 8]. Bisher gibt es jedoch noch keinen In-vivo-Nachweis dieser Kreuzreaktivität [9]. Neben Varizellen oder SARS-CoV-2 können aber auch andere Erreger wie Zytomegalie- oder Herpesviren Autoimmunreaktionen auslösen. Zusammenfassend können Veränderungen des Immunsystems als Folge einer Infektion einen Krankheitsschub eines okulären Schleimhautpemphigoids bedingen.

Schlussendlich kann das Krankheitsbild des OSP nicht geheilt werden. Die konsequente Anwendung der Lokal- und v. a. aber der Systemtherapie sind bei Reaktivierung des Krankheitsbilds dabei für den Erhalt des Sehvermögens essenziell. Operative Interventionen sollten zurückhaltend eingesetzt werden, sind aber bei mechanischen Reizen zur Stabilisierung notwendig. Eine mögliche Therapieoption stellt hierbei die Fornixexpansion mittels AMT und nahtfixierter Illig-Schale dar.

## Fazit für die Praxis

- Patienten mit OSP stellen unter immunsuppressiver Therapie eine Hochrisikogruppe für Infektionskrankheiten dar.
- Die konsequente Anwendung der Lokal- und Systemtherapie sind bei Reaktivierung des Krankheitsbilds für den Erhalt des Sehvermögens essenziell.
- Operative Interventionen sollten zurückhaltend eingesetzt werden, sind aber bei mechanischen Reizen zur Stabilisierung notwendig.
- Eine mögliche operative Versorgung betroffener OSP-Patienten stellt die Fornixexpansion samt Amnionmembrantransplantation, modifiziert mit einer nahtfixierten Illig-Schale, dar.

## References

[1] Wagner H-J (1962). Zur chirurgischen Behandlung des Oberlidnarbenentropiums mit Trichiasis. Klin Monatsblätter Augenheilkd.

[2] Foster CS (1986). Cicatricial pemphigoid. Trans Am Ophthalmol Soc.

[3] De Medeiros VLS, Monteiro-Neto AU, França DDT (2021). Pemphigus vulgaris after COVID-19: a case of induced autoimmunity. Sn Compr Clin Med.

[4] Saha M, Black M (2008). Brit J Dermatol.

[5] Shah S, Danda D, Kavadichanda C (2020). Autoimmune and rheumatic musculoskeletal diseases as a consequence of SARS-CoV-2 infection and its treatment. Rheumatol Int.

[6] Egan CA, Lazarova Z, Darling TN, Yee C, Cote T, Yancey KB (2001). Anti-epiligrin cicatricial pemphigoid and relative risk for cancer. Lancet.

[7] Kamiya K, Aoyama Y, Suzuki T, Niwa H, Horio A, Nishio E, Tokura Y (2016). Possible enhancement of BP 180 autoantibody production by herpes zoster. J Dermatol.

[8] Lucchese A, Di Stasio D, Borgia R, Scivetti M, Petruzzi M (2022). Peptide sharing between SARS-CoV‑2 and bullous pemphigoid 180. J Biol Regul Homeost Agents.

[9] Kasperkiewicz M, Bednarek M, Tukaj S (2021). Case report: circulating anti-SARS-CoV-2 antibodies do not cross-react with pemphigus or pemphigoid autoantigens. Front Med.

